# RETRACTION: The Risk of Intracranial Infection in Adults With Hydrocephalus After Ventriculoperitoneal Shunt Surgery: A Retrospective Study

**DOI:** 10.1111/iwj.70639

**Published:** 2025-04-23

## Abstract

**RETRACTION:** YangY.‐N. , ZhangJ. , GuZ. , and SongY.‐L. , “The Risk of Intracranial Infection in Adults With Hydrocephalus After Ventriculoperitoneal Shunt Surgery: A Retrospective Study,” International Wound Journal 17, no. 3 (2020): 722–728, 10.1111/iwj.13331.32073232 PMC7949404

The above article, published online on 19 February 2020, in Wiley Online Library (http://onlinelibrary.wiley.com/), has been retracted by agreement between the journal Editor in Chief, Professor Keith Harding; and John Wiley & Sons Ltd. Following an investigation by the publisher, all parties have concluded that this article was accepted solely on the basis of a compromised peer review process. The editors have therefore decided to retract the article. The authors did not respond to our notice regarding the retraction.

**RETRACTION:** Y.‐N. Yang , J. Zhang , Z. Gu , and Y.‐L. Song , “The Risk of Intracranial Infection in Adults With Hydrocephalus After Ventriculoperitoneal Shunt Surgery: A Retrospective Study,” International Wound Journal 17, no. 3 (2020): 722–728, 10.1111/iwj.13331.32073232 PMC7949404

The above article, published online on 19 February 2020, in Wiley Online Library (http://onlinelibrary.wiley.com/), has been retracted by agreement between the journal Editor in Chief, Professor Keith Harding; and John Wiley & Sons Ltd. Following an investigation by the publisher, all parties have concluded that this article was accepted solely on the basis of a compromised peer review process. The editors have therefore decided to retract the article. The authors did not respond to our notice regarding the retraction.

